# Development and Validation of Two Instruments Measuring Intrinsic, Extraneous, and Germane Cognitive Load

**DOI:** 10.3389/fpsyg.2017.01997

**Published:** 2017-11-16

**Authors:** Melina Klepsch, Florian Schmitz, Tina Seufert

**Affiliations:** ^1^Department Learning and Instruction, Institute of Psychology and Education, Ulm University, Ulm, Germany; ^2^Department Individual Differences and Psychological Assessment, Institute of Psychology and Education, Ulm University, Ulm, Germany

**Keywords:** Cognitive Load Theory, differentiated measurement, instructional design, multimedia research, multimedia design principles

## Abstract

Cognitive Load Theory is one of the most powerful research frameworks in educational research. Beside theoretical discussions about the conceptual parts of cognitive load, the main challenge within this framework is that there is still no measurement instrument for the different aspects of cognitive load, namely intrinsic, extraneous, and germane cognitive load. Hence, the goal of this paper is to develop a differentiated measurement of cognitive load. In Study 1 (*N* = 97), we developed and analyzed two strategies to measure cognitive load in a differentiated way: (1) Informed rating: We trained learners in differentiating the concepts of cognitive load, so that they could rate them in an informed way. They were asked then to rate 24 different learning situations or learning materials related to either high or low intrinsic, extraneous, or germane load. (2) Naïve rating: For this type of rating of cognitive load we developed a questionnaire with two to three items for each type of load. With this questionnaire, the same learning situations had to be rated. In the second study (*N* = between 65 and 95 for each task), we improved the instrument for the naïve rating. For each study, we analyzed whether the instruments are reliable and valid, for Study 1, we also checked for comparability of the two measurement strategies. In Study 2, we conducted a simultaneous scenario based factor analysis. The informed rating seems to be a promising strategy to assess the different aspects of cognitive load, but it seems not economic and feasible for larger studies and a standardized training would be necessary. The improved version of the naïve rating turned out to be a useful, feasible, and reliable instrument. Ongoing studies analyze the conceptual validity of this measurement with up to now promising results.

## Introduction and Theoretical Background

One of the most powerful and most debated frameworks in educational research during the last few decades has been Cognitive Load Theory (CLT; Sweller and Chandler, 1991; Sweller, 2010a), which is extensively used for evaluating learning environments or interpreting empirical results. However, the absence of an adequate measure of cognitive load has been criticized (Moreno, 2010). The vast majority of studies on multimedia learning assess cognitive load by using a single item to assess perceived invested mental effort (Paas, 1992). Some other studies used objective techniques, such as dual task measures (e.g., Brünken et al., 2004), or physiological parameters (e.g., heart rate: Paas and van Merriënboer, 1994; eye blink parameters: Goldstein et al., 1992). All these measures show different strengths and weaknesses as will be discussed below. However, until now there has been no instrument that allows measuring the three types of load differentially, namely intrinsic (ICL), extraneous (ECL), and germane cognitive load (GCL) (Brünken et al., 2010; Moreno and Park, 2010). Such a differentiated measure would help to improve our understanding of which aspect of the learning task requires cognitive resources to gain insight into the learning processes and the effects of instructional design.

### Cognitive Load Theory (CLT)

In a learning situation, information must be processed in working memory and stored in long-term memory. One of the main assumptions of CLT is that working memory is limited in capacity, when processing information, as well as in time, when it comes to holding information (Paas et al., 2010). The second assumption of CLT is that long-term memory is virtually unlimited (Sweller et al., 1998), and, according to schema theory, knowledge is organized and retained in the form of schemata (Rumelhart, 1981).

The merit of CLT is to make prescriptions for instructional design that reflect these specific characteristics of human cognitive architecture. Following these prescriptions, successful instruction should allow learners to manage working memory load and, hence, to learn successfully. For a long time, CLT differentiated three independent sources of memory load, namely ICL, ECL, and GCL (e.g., Sweller et al., 1998). While intrinsic load arises from the number of interrelated elements of the learning task, extraneous load is caused by the additional requirements that are produced by suboptimal instructional design and are not directly linked to the task. The third source of memory load is germane load, which “reflects the effort that contributes to the construction of schemas” (Sweller et al., 1998, p. 259). All three aspects will be described in the following paragraphs as they inspired the construction of the developed instruments.

*Intrinsic cognitive load* (Sweller, 1994; Sweller and Chandler, 1994) is the load resulting from the inherent complexity of the learning task. This load type depends on two different factors (Moreno and Park, 2010), the element interactivity of the task and the learner’s prior knowledge. (1) *Element interactivity* (Chandler and Sweller, 1996) corresponds to the number of elements that the learner must simultaneously process in working memory while dealing with the task. Low element interactivity, therefore, means that a learner can process the elements sequentially as there is only minimal reference between the elements, whereas tasks with high element interactivity comprise elements that are highly interlinked and must, therefore, be processed simultaneously (Sweller, 2010b). The intrinsic load in language learning is, for example, low when you have to repeat unrelated vocabularies instead of constructing sentences by using the correct grammar. (2) The *prior knowledge* of the learner plays a fundamental role as new information can be linked with existing schemata (Gerjets et al., 2004). Hence, the information that must be processed is more comprehensive and well structured so learners do not have to process (too) many unrelated elements in working memory. A typical example is an expert in chess who can build meaningful chunks instead of memorizing many unrelated elements. According to these two factors, two approaches to manipulate or reduce ICL, have been empirically tested: (1) The *segmenting principle* (Mayer and Moreno, 2010) aims at reducing element interactivity by presenting information step by step, which helps learners with insufficient prior knowledge to organize the incoming information. So with each step, they gain more prior knowledge and are able to better link the incoming information of the next step. The segmentation into steps is a matter of design and can be done by presenting information in parts, for example on different sheets or in the formatting of paragraphs. This describes why segmenting may also reduce ECL. (2) The *pretraining principle* (Mayer, 2005a) reduces ICL by providing the learner with information about the content before starting with the learning material. Increasing the learner’s prior knowledge supports the integration of new information. In general, it is difficult to substantially reduce intrinsic load without altering the learning objectives. It is usually not possible to reduce information or complexity without altering the task itself by skipping something. When students have to understand a specific issue, they must face all the relevant ideas and their inherent structure. Therefore, only altering the task itself, e.g., by adding or deleting information or having learners with different levels of prior knowledge, results in a real variation of ICL and not a “virtual” reduction of ICL because of design effects. As one may notice, trying to change ICL can easily end up in also changing ECL. Hence, most instructional designers do not concentrate on what must be learned but on how to present the necessary information and therefore, on ECL.

*Extraneous cognitive load* is caused by the instructional design of the learning material. Whenever the learner must invest mental resources into processes that are not relevant for the task itself, like searching for or repressing information, we call them extraneous processes. Consequently, ECL is reduced when necessary processes, such as imagery or linking information, is facilitated by design. This means that the designer of the learning material can manipulate ECL in a relatively easy way.

Hence, many researchers addressed the question of how to reduce ECL in a reasonable way. They found vast empirical evidence for such effects, also called multimedia design principles. A few of the principles, which we refer to in the following studies, are included herein. (1) The *multimedia principle* (Mayer, 2001; Fletcher and Tobias, 2005): numerous empirical findings show that learners perform better when learning from a combination of text and pictures rather than from pictures alone. Nevertheless, there are some boundary conditions to this principle, as described in a variety of other principles (e.g., the *redundancy principle*: Sweller and Chandler, 1994 or the *coherence principle*: see below). (2) The *modality principle* (Mayer and Moreno, 2003; Low and Sweller, 2005; Mayer, 2005a): According to this principle it is better to present a text auditorily, not visually, when combined with a picture. While printed text combined with a picture requires visual resources, the auditory text uses the phonological system of working memory (Baddeley, 2003). Since this system is independent from the visual system, the learner can process text and picture simultaneously, which helps to integrate the two sources. So, working memory capacity is used more effectively, and an overload of the visual capacity is prevented. (3) The *coherence principle* (Mayer and Moreno, 2010): This principle recommends leaving out all decorative information in multimedia learning environments that does not contribute to the learning task itself. For example, decorative pictures may be motivating, but they distract attention and need to be disregarded as they are not relevant for learning (as a connected concept, see also *seductive details effect*: Rey, 2012). (4) The *split attention effect* (Ayres and Sweller, 2005): Split attention occurs when learners are forced to mentally integrate separated sources of information that could be better presented in an integrated way. This effect is also known as the *spatial contiguity principle* (Mayer and Moreno, 2010), which occurs when learners benefit from a spatial integration, e.g., when text information is presented within instead of beside a picture. The integration could also take place timely (*temporal contiguity principle*: Mayer and Moreno, 2010), so text information accompanying dynamic pictures like video or animation should be presented simultaneously instead of delayed. The integrated, or simultaneous, presentation facilitates synchronous mental processing and, hence, the integration of information.

Overall, there are many ways to optimize instructional design to reduce ECL (for more principles/effects, see Mayer, 2005b; Sweller, 2010a). With the freed-up resources, learners could invest more effort into in-depth learning processes.

*Germane cognitive load*, the third type of cognitive load, results from activities required of a learner that facilitate learning and contribute to transfer performance, helping to build correct mental models (Paas et al., 2003a). Such activities could be, for example, taking notes during reading a text, coming up with memory hooks to remember something, or explaining the learned content to someone else. Therefore, high germane load indicates that learners are engaged and direct their mental resources to learning processes. Researchers also addressed the question of how to foster this type of load (e.g., Paas and van Gog, 2006) and described several design principles. In the following studies, we refer to the *self-explanation effect* (VanLehn et al., 1992) for varying GCL. This effect indicates that learners who explain learning material to themselves achieve a higher learning outcome. They actively link the new information with existing schemata and, hence, invest in deeper learning processes. In our studies, we used different approaches to induce self-explanation. We, for example, asked learners to engage in different learning activities, e.g., to formulate titles to text paragraphs, to find examples to a topic, or to sum up a topic in their own words. Other strategies have been to engage memory strategies through prompts (Berthold et al., 2007, 2009).

The given descriptions make it obvious that germane load is highly dependent on intrinsic load. Moreover, learners are only able to devote germane resources if the amount of extraneous load is not exceeding their working memory capacity. Hence, germane load is also linked to extraneous load. Therefore, an attempt was made to refine CLT and conceptually differentiate those aspects of load that are either intrinsic to the task, and therefore productive, or those that are extraneous, and therefore unproductive. Hence, a new definition of germane load is that it “refers to the working memory resources available to deal with the element interactivity associated with ICL” (Sweller, 2010b, p. 126). From a theoretical point of view, this clarification is helpful and necessary. From a measurement point of view, it is nevertheless relevant to understand all loading aspects in a learning situation: the resources required by the task or those resources available that are deliberately devoted by the learner. Therefore, we address all aspects of cognitive load: the intrinsic and productive aspects, including the given intrinsic element interactivity and the devoted germane resources, to understand these interactions and the extraneous and unproductive load. Given the three-partite nature of cognitive load, *ICL*, *ECL*, and *GCL* all need to be considered, as all of them are important and noteworthy when generating and designing learning material. However, to date, there has been no instrument that allows for the assessment of all three types of load in a differentiated way.

### Cognitive Load Measurement

Many researchers stated that measuring cognitive load is one of the persistent challenges in educational research (Mayer and Moreno, 2002; Brünken et al., 2003, 2010; Schnotz and Kürschner, 2007; de Jong, 2010; Moreno, 2010). It has even been questioned whether it is a “mission impossible” (Brünken et al., 2010) facing the measurement approaches at hand. Those approaches that use either subjective ratings or objective measures, such as dual-task performance, are directly addressing load, e.g., by asking learners to rate their perceived mental load, or indirectly by using indicators that are connected to load, such as performance measures. Moreover, they are either online and measure constantly or repeatedly during the learning process or offline and measure retrospectively. All these measures show strengths and weaknesses (see Brünken et al., 2003, 2010). In the following pages, we will describe and briefly discuss the most common measurement approaches: (1) self-report measures, (2) dual-task measures, and (3) measures of physiological parameters.

#### Self-report Measures

The most popular scale for measuring cognitive load is a rating scale developed by Paas (1992). This scale is a modified version of a scale by Bratfisch et al. (1972), which was constructed to measure task difficulty. In fact, the scale used by Paas consists of one item, and participants are asked to use a 9-point Likert scale for their responses, ranging from very, very low mental effort (1) to very, very high mental effort (9). The exact wording of the item is not published in the article, but it is similar to ratings used by many other researchers (for an overview, see Paas et al., 2003b). Typical item wordings: “I invested … mental effort” or “my invested mental effort was ….” The scale is usually designed as a 5- to 9-point Likert scale. While one-item scales appear to be economic at first glance, they are generally problematic from a psychometric perspective. There is no way to tease apart true variance from measurement error. This is a problem discussed in short by van Gog and Paas (2008). They complain about different wordings, different labels, and an inconsistent scale range. The fact that introspective ratings are not highly reliable has been demonstrated by the study of van Gog et al. (2011), where overall retrospective measures of load were generally higher than the mean of several measures during learning. Learners adjust their ratings with respect to situational parameters, and they use subjective internal standards to evaluate their current load state, if they even possess the ability of introspection.

More importantly, one item cannot distinguish between the various sources of load postulated in CLT. In fact, sources of load have been inferred from the combination of task performance with rated mental effort in some previous studies: If learning outcome is low, despite highly rated mental effort, this is interpreted as resulting from inappropriate affordances of the learning materials (i.e., extraneous load). But, high task difficulty (intrinsic load) offers a plausible explanation too. However, such inferences from outcome likely result in a circular argument (Kalyuga, 2011; Kirschner et al., 2011) and should be avoided.

A second frequently used measurement technique is a one-item rating of task difficulty (DeLeeuw and Mayer, 2008; for an overview, see van Gog and Paas, 2008 or Brünken et al., 2010). Overall, its limitations are the same as for the mental-effort rating. For a broader overview on studies using one-item subjective cognitive load measures, also see Paas et al. (2003b).

For self-report measures that measure different aspects of CLT see section “Measuring Cognitive Load in a Differentiated Way”.

#### Dual-Task Measures

Cognitive load measures using the dual-task paradigm require a learner to perform two tasks simultaneously. It is assumed that performance for the second task drops when the primary task, i.e., the learning task, becomes more loading. There are two possible ways to conduct dual-task measures. (1) On the one hand, it is possible to measure accuracy and response times in an observation task that needs to be carried out during the performance of the learning task (e.g., Brünken et al., 2002). (2) On the other hand, a concurrent second task needs to be performed during learning (e.g., Park and Brünken, 2011). This, for example, could be tapping your feet in a given rhythm as constant as possible. Increasing load in the first task could then be measured by impairments of the secondary tasks. Dual-task measures of load have the advantage of being objective, and they mirror the whole learning process so you can gather rich data. However, the most obvious disadvantage is the intrusiveness of such techniques; they disturb the learning process and impose load by themselves. Besides it is a matter of resources: Learners with high working memory capacity might not be as loaded by a secondary task as learners with low working memory capacity. This would always result in the need to control the prerequisites of learners associated with working memory. Another disadvantage is, as already mentioned for the subjective ratings, that it is also not possible to identify the type of load that is measured.

#### Measures of Physiological Parameters

A wide range of physiological parameters have been used as indicators for cognitive load. The most commonly used physiological parameters are heart rate (Paas and van Merriënboer, 1994), pupil dilation (van Gerven et al., 2004), and electroencephalography measures (Antonenko et al., 2010). There are also some less common ones, such as measuring hormone levels (Wilson and Eggemeier, 1991) or using fMRI measures (Whelan, 2007). However, it is difficult to tell what triggered the physiological processes and, hence, to interpret the data (Brünken et al., 2010). Moreover, the measures are usually intrusive and less economic, and the problem is again that it is impossible to tell if ICL, ECL, or GCL is being measured.

### Measuring Cognitive Load in a Differentiated Way

A major shortcoming of the previously discussed approaches is that they only assess the overall amount of experienced load and do not distinguish between intrinsic, germane, or extraneous load. This limits their usefulness in instructional design and multimedia learning research that build on the differentiated CLT. To overcome this limitation, some researchers have developed questionnaires to measure cognitive load in a differentiated way.

Several researchers (e.g., Gerjets et al., 2006; Zumbach and Mohraz, 2008) used variations of the NASA Task Load Index (Hart and Staveland, 1988) in an attempt to measure cognitive load in a differentiated way. However, the wordings of those variations are not always well documented. Also, the study of Naismith et al. (2015), in which they compare their questionnaire called cognitive load component (CLC) with the NASA-TLX also does not provide sufficient information on the items and scales.

Cierniak et al. (2009) and Cierniak (2011) used three items to represent ICL, ECL, and GCL. In their studies, they found significant matches between performance data and measured cognitive load. They asked for “difficulty of the learning content” (adopted from Ayres, 2006) to represent ICL, “difficulty to learn with the material” (adopted from Kalyuga et al., 1998) to represent ECL, and “concentration during learning” (adopted from Salomon, 1984) to represent GCL. However, they didn’t test their questions with a wide variety of learning material to validate their scale.

Swaak and de Jong (2001) used a measurement scale called SOS to measures participants’ cognitive load during learning in a simulation environment about electrical circuits. In a first version, they used three items to measure difficulty of the subject, difficulty of working with the operating system, and usability of support tools. It was not explicitly specified which item refers to which type of load. All three items had to be answered on a scale ranging from “extremely easy” (0) to “extremely difficult” (100). They did not find differences in cognitive load in their study between the experimental groups. An adapted and extended version of the SOS scale was used by Eysink et al. (2009) in multimedia learning arrangements. ICL was measured by asking for the perceived difficulty of the domain. ECL was measured using three items asking for accessibility of information (collect all needed information), design (distinguishing important from unimportant information), and navigation (working with the learning environment). GCL was measured by asking for the difficulty of understanding the simulation. They found differences for all three types of cognitive load, but they were not theoretically linked or discussed regarding the learning outcome, i.e., productive or unproductive aspects.

One of the most recent approaches to measure cognitive load differentially that has attracted attention in the field is a scale by Leppink et al. (2013). They developed a questionnaire for complex knowledge domains consisting of 10 items, which were tested in the domain of statistics. The questionnaire included three items on ICL that asked about the complexity of the topics, formulas, and concepts of the activity. It included three items on ECL that refer to the instruction and/or explanations given during the activity and asked, e.g., about ineffectivity. For GCL four items were included that referred to enhancement of understanding of the topics, formulas, concepts, and definitions. One of the GCL items particularly referred to the understanding of statistics. According to the authors, the term *statistics* could be replaced by any other knowledge domain. They found promising results and tried to replicate their findings in another set of experiments (Leppink et al., 2014). In Study 1, they replicated the factor structure in a similar context (statistics education) but within a different context (language learning). Even if they had to adopt the items to fit the different domains, they found the three factors to be robust. In Study 2, they again adopted the items to fit the domain and added three more items explicitly asking for “invested mental effort” on the three types of load. In their studies, they were missing a positive correlation between items that are supposed to measure GCL and learning outcomes as along with a substantial correlation between the “old” items and the new one. As a conclusion, according to Sweller et al. (2011), they only approved the measurement of intrinsic and extraneous load as being meaningful.

Based on these previous attempts to measure cognitive load differentially, we developed and evaluated self-report measures that tap the different aspects of cognitive load in a differentiated way. We decided on a self-reported measure of load due to its economy and flexibility. Our goal was to develop a reliable domain-unspecific questionnaire that could be validated and used in various learning situations. Whereas measures of ECL and ICL within our questionnaire should evaluate the inherent complexity and the design of the learning material as it was perceived by the learner (based on his prior knowledge and expertise and his prerequisites for different instructional designs) during learning, measures of GCL should focus on the additional investment of cognitive processes into learning (triggered through elements that learning material contains, e.g., prompts). The most straightforward way to address this challenge was to develop a self-report questionnaire—which we will refer to as naïve rating in the following. Items would reflect all three aspects of cognitive load. We additionally followed another path, in which we qualified students to understand and differentiate the three aspects of cognitive load—we will refer to this as an informed rating in the future. The questionnaire in this case asked, without paraphrasing, to rate the intrinsic, extraneous, and germane load for each learning scenario.

To analyze whether the two approaches to measure cognitive load in a differentiated way are reliable, valid, and comparable, we conducted two experimental studies. In our first study, we were interested in the comparability and quality of the two instruments. In the second study—with an additional pilot-study—we focused on the refinement of the questionnaire for the naïve rating and, hence, on developing a new, economic, domain-independent, and especially differentiated measure of cognitive load.

## Study 1

This first study was conducted to compare the two approaches to measure cognitive load, the informed rating and the questionnaire for a rating without prior knowledge about the concepts of load, which we, therefore, call a naïve rating. We could have used a learning scenario in which learners carry out a learning task and rate their experienced load in this situation afterward, as most other researchers have chosen to do (e.g., Cierniak et al., 2009; Leppink et al., 2013). To evaluate whether our instruments could detect differences—in the amount of load and in the type of load—in multiple different learning situations, we used descriptions of hypothetical learning situations (verbally or in pictorial form via screenshots). Each scenario had to be evaluated by means of the respective questionnaires. Therefore, learning situations and load rating were hypothetical. However, one strength of this approach was that it allowed us to cross-validate our measures in various settings and to compare our two instruments for several ratings. Additionally, we analyzed the reliability of our scales.

### Methods and Materials

#### Participants and Design

The participants in Study 1 included 97 computer science or psychology students from a German university in their first or third semester. No other demographic data were assessed. Each participant was randomly assigned to the informed rating group (*n* = 48) or the naïve rating group (*n* = 49). Dependent variables were ICL, ECL, and GCL for each task, either measured by the informed or naïve questionnaire.

#### Procedure

At the beginning, all participants were informed about the procedure and signed an informed consent, and participants were aware that they could withdraw their data at any point in the study. Afterward, each participant was randomly assigned to one of the two groups: (1) The informed rating group, which got an introduction to CLT, as described later, to be able to rate the following tasks in an informed way, and (2) the naïve rating group, whose members did not get any previous information about CLT but started directly with the evaluation tasks. The evaluation tasks, as described below, were small learning tasks or scenarios which had to be conducted. Participants also were asked to write down the correct answer, if possible. After each task, each participant filled out either the informed rating questionnaire or the naïve rating questionnaire, both described below. Altogether, the study took about 90 min for the informed rating group and 60 min for the naïve rating group.

#### Evaluation Tasks

For this experimental study, we developed 24 learning tasks or scenarios grouped in five different domains (language learning, biology, mathematics, technology, and didactics). In Study 1, tasks within each domain varied in only one aspect of cognitive load (ICL, ECL, or GCL), following from theoretical accounts of CLT, empirical results of cognitive load related studies, or the above-mentioned multimedia design principles. All evaluation tasks are displayed in Supplementary Table S1. It should be mentioned that we quickly noticed that the implementation of a variation of one type of load did not affect another type of load. Learners had to rate all three types of cognitive load for all these tasks/scenarios. Here are some examples of variation: (1) For ICL, learners had to rate cognitive load for a task where the element-interactivity had been varied, e.g., “the day after tomorrow will be Saturday. Which day was yesterday?” which was supposed to be rated with lower ICL scores versus “3 days after yesterday was Friday. Which day will be 5 days before tomorrow?” where the ICL should be rated higher. In the vocabulary tasks, we used, for example, languages with different inherent complexity. (2) For extraneous load, we showed learning environments with an integrated format of text and picture (lower ECL) versus a separated format (higher ECL). Another variation we used was material with (high extraneous load) or without seductive details (low extraneous load). (3) For germane load, we asked participants to rate different instructional settings that should either induce GCL, like “every 20 min a teacher gives you time to think of examples you can find for the topic” versus tasks without such an activation. Nevertheless, learners had to rate all three types of load after conducting each task to examine whether the ratings in fact only differed with respect to the theoretically assumed type of load. Everyone was also asked to write down the correct answer for the given learning task. For the learning scenarios used for variations of GCL which had to be merely imagined, nothing had to be written down.

#### Cognitive Load Measures

##### Informed rating

If learners know what these load types are and in which way they differ, they should be able to rate the three types of cognitive load correctly. Thus, we first developed an introduction into CLT. The introduction included information about CLT itself, working memory, and types of load. It was presented as a lecture using PowerPoint slides. The lecture ended with a few examples, showing how variations of the three types of load could be implemented in typical learning materials. These examples were different from the tasks to be rated. The design principles and domains used were sometimes overlapping, but the tasks themselves were completely new to the participants to prevent replicating information from the lecture. The aim was to qualify the participants to detect the three types of load and in which ways they are interrelated and how they can be differentiated from each other. The whole training, including a discussion, lasted about 30 min. Afterward the training, the participants might not be experts, but they should be able to rate cognitive load differentially in an informed way. After the training, we handed out a written summary of the three types of cognitive load. This little booklet was allowed during rating, so our participants were able to look up the types of load during rating if they did not feel confident with the concepts of load. The developed questionnaire for informed rating directly targets the three types of load. Therefore, we developed three items, which read as follows: (1) “During this task, ICL was…,” (2) “During this task, ECL was…,” and (3) “During this task, GCL was….” As a fourth question, the informed rating questionnaire included a question about the overall mental load during the learning situation that was adopted from Paas (1992). All items had to be rated on a 7-point Likert scale from “very low” to “very high.”

##### Naïve rating

The other group of participants was not informed about the concept of cognitive load and, therefore, rated the same learning situations in a naïve way by completing the self-report questionnaire. This first version comprised two questions related to ICL, three related to ECL, and another two items related to GCL. All items had to be rated on 7-point Likert scales from “completely wrong” to “absolutely right.” As an eighth question, the naïve rating included the same question as the informed rating about the overall mental load during the learning situation. All items for the naïve rating are shown in **Table 1**. The items of the questionnaire were developed in German and were translated into English for this paper. An advantage of this approach is that it does not require an introduction into CLT. However, the aim was to find items that are very clear, so participants would easily understand the questions; also, the questions needed to be clearly related with the respective sources of cognitive load.

**Table 1:** Items of the first version of the naïve rating questionnaire.

Type of load	Item - German	Item - English
ICL	Bei der Aufgabe musste man viele Dinge gleichzeitig im Kopf bearbeiten.	For this task, many things needed to be kept in mind simultaneously.
ICL	Diese Aufgabe war sehr komplex.	This task was very complex.
GCL	Bei dieser Aufgabe musste ich selbst ganz aktiv nachdenken.	For this task, I had to highly engage myself.
GCL	Bei dieser Aufgabe musste ich intensiv überlegen, wie einzelne Dinge gemeint sind.	For this task, I had to think intensively what things meant.
ECL	Bei dieser Aufgabe ist es mühsam, die wichtigsten Informationen zu erkennen.	During this task, it was exhausting to find the important information.
ECL	Die Darstellung bei dieser Aufgabe ist ungünstig, um wirklich etwas zu lernen.	The design of this task was very inconvenient for learning.
ECL	Bei dieser Aufgabe ist es schwer, die zentralen Inhalte miteinander in Verbindung zu bringen.	During this task, it was difficult to recognize and link the crucial information.


*ICL, intrinsic cognitive load; ECL, extraneous cognitive load; GCL, germane cognitive load.*

#### Data Analysis

*Reliability* was analyzed separately for both instruments. To this end, we calculated internal consistency for each of the 24 tasks. For the *informed rating*, there was only one item per load type. Consequently, we analyzed whether the three load types form one single construct, or—as we would expect—three separable constructs, i.e., internal consistency of all three items together should be rather low. To report this data, a meta-analysis of coefficient alpha based on formulas presented by Rodriguez and Maeda (2006) has been conducted, which allows us to conduct a mean of several given alphas based on sampling distribution. For the *naïve ratings*, there were several items for each load type, and internal consistency could be computed for the items of each scale (separately for each task), and this was predicted to be high. Again, to report this data in an aggregated way, the before-mentioned weighted mean was conducted to generalize Cronbach’s alpha. For the naïve rating, we also calculated internal consistency for all seven items and aggregated them.

*Validity* was analyzed by comparing the learners’ ratings with the theoretically predicted outcome, i.e., whether the participants rated the specific loads as either low or high, according to our intended task design. Therefore, tasks with differing load levels should be rated significantly different in the amount of this specific load. This means, e.g., all tasks developed to induce low intrinsic load should be rated significantly lower than all tasks developed to induce high intrinsic load.

To *compare the two instruments*, we compared means by a *t*-test. This should reveal, whether there are significant differences between the scales for ICL, ECL, and GCL of the informed and naïve ratings. We expected the two ratings not to differ and followed a conservative approach, when we decided that we accept the ratings not to be significantly different if *p* > 0.20^1^.

Comparability and validity will be analyzed by means of a mixed analysis of variance (ANOVA) for each type of load.

Finally, relations with the established global load measure by Paas (1992) were investigated. Each participant in the informed and the naïve rating group also filled out this scale for each task. For the informed rating, we correlated the three items separately for the mental effort item. For the naïve rating, we correlated our three load-type scales for the mental effort item. We predicted moderate to high correlations, as mental effort should be rated high whenever a task implies a high load—irrespective of which load type—and should be low whenever a task is designed to result in low load. Therefore, for both ratings we also report correlations of the sum of the three load ratings with the mental effort rating.

### Results

The *reliability* of the informed rating test depended on tasks. For each task, we analyzed the internal consistency of the ICL, ECL, and GCL item. As expected, reliability was low for all the tasks. Reliability generalization of Cronbach’s α resulted in a low α = 0.25, as expected due to the different aspects of load which we measured with the three items.

For the naïve rating, we first conducted the reliability test for the two, respectively, three items for each load type for each task. The aggregated alphas that we obtained though reliability generalization have been as expected: For ICL α = 0.86, for ECL α = 0.80, and for GCL α = 0.80. The internal consistency between all ICL, ECL, and GCL items (seven items) for each task in the naïve rating showed an aggregated α of 0.86, which was way higher than expected.

A mixed ANOVA for each type of load was used to *compare the two questionnaires* (naïve vs. informed rating – between subjects) and to *validate* if the rated amount of load between high and low load tasks differs for both questionnaires in general (low vs. high load tasks – within subjects). We also calculated contrasts for each questionnaire between high and low load tasks and contrasts for the differing amount of load between the two instruments.

**FIGURE 1:**
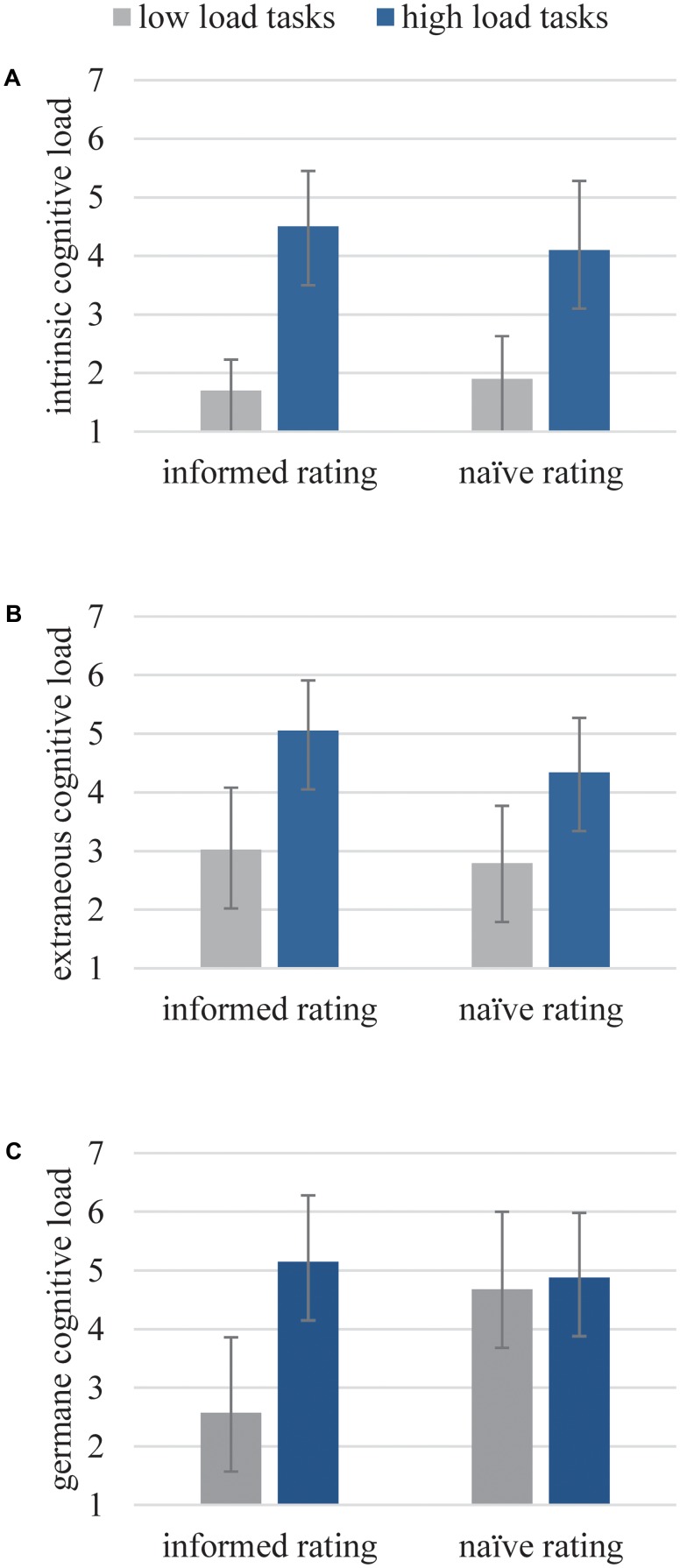
Subjective ratings of the informed and naïve raters for ICL **(A)**, ECL **(B)**, and GCL **(C)** for corresponding theoretical low or high load tasks.

For ICL (see **Figure 1A** and **Table 2**), we found a main effect for the amount of load [*F*(1,89) = 490.23, *p* < 0.001, η^2^ = 0.85]. Theoretically low ICL tasks (*M* = 1.80, *SD* = 0.64) have been rated significantly lower than theoretically high ICL tasks (*M* = 4.29, *SD* = 1.09) with both instruments. Concerning the validity of the two instruments, respectively, we also found a significant difference between low and high load ratings for each instrument [informed rating: *F*(1,89) = 293.32, *p* < 0.001, η^2^ = 0.77; naïve rating: *F*(1,89) = 199.96, *p* < 0.001, η^2^ = 0.69]. For comparability, no main effect, i.e., no significant difference between the two approaches, has been found [*F*(1,89) = 1.18, *p* = 0.28, η^2^ = 0.01]. Contrasts support this result [low ICL tasks: *F*(1,89) = 0.79, *p* = 0.38, η^2^ = 0.01; high ICL tasks: *F*(1,89) = 3.81, *p* = 0.05, η^2^ = 0.04]. An interaction effect could be found [*F*(1,89) = 6.13, *p* < 0.05, η^2^ = 0.06] because the informed raters rated more extreme than the naïve raters.

**Table 2:** Means (M) and standard deviations (SD) for subjective ratings of the informed and naïve raters for ICL, ECL, and GCL for corresponding theoretical low or high load tasks.

Type of load		Informed raters		Naïve raters	
			
		Low load tasks	High load tasks	Low load tasks	High load tasks
ICL	*M (SD)*	1.74 (0.53)	4.52 (0.95)	1.86 (0.73)	4.08 (1.18)
ECL	*M (SD)*	3.02 (1.06)	5.05 (0.86)	2.79 (0.98)	4.34 (0.93)
GCL	*M (SD)*	2.57 (1.29)	5.15 (1.13)	4.68 (1.32)	4.88 (1.10)


*ICL, intrinsic cognitive load; ECL, extraneous cognitive load; GCL, germane cognitive load.*

For ECL (see **Figure 1B** and **Table 2**), we also found a main effect for the amount of load [*F*(1,95) = 275.91, *p* < 0.001, η^2^ = 0.74]. Theoretically low ECL tasks (*M* = 2.91, *SD* = 1.02) have been rated significantly lower than theoretically high ECL tasks (*M* = 4.69, *SD* = 0.96) across instruments. Contrasts reveal the same results for each questionnaire [informed rating: *F*(1,95) = 174.60, *p* < 0.001, η^2^ = 0.65; naïve rating: *F*(1,95) = 105.32, *p* < 0.001, η^2^ = 0.53]. For comparability, a main effect has been found [*F*(1,95) = 8.32, *p* < 0.05, η^2^ = 0.08]: the informed raters reported higher ECL ratings (low ECL tasks: *M* = 3.03, *SD* = 1.06; high ECL tasks: *M* = 5.05, *SD* = 0.86) than the naïve raters (low ECL tasks: *M* = 2.79, *SD* = 0.98; high ECL tasks: *M* = 4.34, *SD* = 0.93). Contrasts revealed no significantly different ratings between the questionnaires for theoretically low ECL tasks [*F*(1,95) = 1.30, *p* = 0.26, η^2^ = 0.01] but different ratings for theoretically high ECL tasks [*F*(1,95) = 14.92, *p* < 0.001, η^2^ = 0.14]. The interaction effect is also significant [*F*(1,95) = 4.72, *p* < 0.05, η^2^ = 0.05].

For GCL (see **Figure 1C** and **Table 2**), we again found a main effect for the amount of load [*F*(1,88) = 53.24, *p* < 0.001, η^2^ = 0.38]. Again, in general, theoretically low GCL load tasks (*M* = 3.67, *SD* = 1.68) have been rated significantly lower than theoretically high GCL tasks (*M* = 4.88, *SD* = 1.13). The same pattern could be found for the informed rating [*F*(1,88) = 105.84, *p* < 0.001, η^2^ = 0.55]. For the naïve rating, no significant difference between theoretically high and low GCL tasks was found [*F*(1,88) = 0.04, *p* = 0.84, η^2^ < 0.001]. For comparability, we found a main effect [*F*(1,88) = 18.02, *p* < 0.001, η^2^ = 0.17]. The rating of theoretically low load tasks differed between questionnaires [*F*(1,88) = 58.66, *p* < 0.001 η^2^ = 0.40] as did the rating for theoretically high load tasks [*F*(1,88) = 4.82, *p* < 0.05, η^2^ = 0.05]. The informed group rated much more sophisticated (low GCL tasks: *M* = 2.57, *SD* = 1.29; high GCL tasks: *M* = 5.14, *SD* = 1.13), whereas the naïve ratings are on a high level for low GCL tasks (*M* = 4.68, *SD* = 1.32) and high GCL tasks (*M* = 4.88, *SD* = 1.10). Again, a significant interaction effect can be found [*F*(1,88) = 57.35, *p* < 0.001, η^2^ = 0.40].

Concerning the question of whether the differentiated items (informed rating), respectively, scales (naïve rating) fit to ratings on the overall mental-effort item correlations for each task were conducted. Also, the sum of the items/scales and their correlation with the global load measure by Paas (1992) were calculated.

We found substantial correlations for almost all ratings of the informed group: For tasks where ICL was varied, we found correlations between the intrinsic-load rating and the mental-effort item of *r* between 0.41 and 0.68 for the respective tasks. If ECL was varied, we found correlations between the extraneous-load rating and the mental-effort item of *r* between 0.32 and 0.60. The germane-load rating correlated with the mental-effort item with *r* between 0.32 and 0.41 in those tasks where GLC has been varied. When adding together the ratings for ICL, ECL, and GCL of the informed raters as a sum and correlating it with the mental-effort item for each task, we got correlations of *r* between 0.32 and 0.69.

For the naïve rating concerning tasks where ICL was varied the correlations of the intrinsic-load rating with the mental-effort item resulted in *r* between 0.47 and 0.81. The correlations of the extraneous-load rating with the mental-effort item was *r* between 0.36 and 0.68 for tasks where ECL was varied. When GCL was varied, we found correlations of the germane-load rating and the mental-effort item of *r* between 0.51 and 0.77. Again, when adding together the ratings for ICL, ECL, and GCL of the naïve raters as a sum and correlating it with the mental effort item for each task, we got correlations of *r* between 0.46 and 0.85.

### Discussion

We analyzed and compared two different approaches to measure the three conceptual parts of cognitive load differentially concerning their validity, i.e., their power to confirm theoretically predicted ratings and their reliability.

Our results first provide evidence that the *informed rating* seems to be a valid method of measuring cognitive load in a differentiated way. Participants were able to rate the amount of different aspects of cognitive load as intended by the design and in line with the theoretical predictions. The fact that the overall reliability was very low suggests that learners perceive different levels of load for the three distinct parts of load; ICL, ECL, and GCL. This is not surprising, given that the three types of load are clearly distinguished in theory (Moreno and Park, 2010). Nevertheless, we should analyze reliability of this measure with other reliability parameters, such as retest-reliability or split half-tests in future studies. Furthermore, as the learners could differentiate between the three types of load and as the independence of the three types seem to be sufficiently given, we argue that an overall measure without differentiating these parts, like the often-used mental-effort scale, is not adequate. To further improve our informed rating, better standardization of the instruction may be indicated. In our case, it was a spoken lecture. A more standardized version of the introduction could instead be given independently from a personal instructor in either written form with a booklet or an interactive e-learning environment. This learning environment should again address all concepts of CLT and contrast learning material with different parameter values of ICL, ECL, and GCL.

The *naïve rating* instrument showed satisfying internal consistency for all three aspects of cognitive load. The scales were also valid for ICL and ECL, but not for GCL. While informed ratings scored germane load of the task as induced, this was not the case for the naïve rating. Learners failed to differentiate between theoretically predicted low and high levels of germane load. Especially problematic was that the naïve ratings for theoretically low GCL tasks were extremely high. Therefore, the current items should be revised to increase the validity of the germane-load scale. It could be speculated that the wording of the current items was ambiguous so learners understood them differently. We should also keep in mind that the informed raters could detect differences in load levels more clearly. The questionnaire also allowed us to differentiate between high and low load levels, albeit it appeared not as sensitive in the present form.

In this study, we designed tasks that should differ in the level of one type of load specifically. However, we cannot design tasks that mirror an exact amount of a special load type. Hence, we cannot qualify the absolute accuracy of the learners’ ratings. Moreover, in a naturalistic learning setting all three types of load can always be qualified as either high or low. In future research, real learning tasks should be employed instead of hypothetical ones. Second, all three types of load should be rated for each task based on theoretical assumptions. Finally, larger samples can be recommended for the development of a questionnaire.

Overall, the measure using informed ratings worked arguably well. However, this strategy is not very economic, and, therefore, difficult to put into practice in educational research. Hence, in our second study, we focused on improving the naïve ratings measure. Given that the items for ICL and ECL functioned well, we focused on improving the measurement of GCL and to analyze again the validity and reliability of our questionnaire.

## Study 2

Based on the results of the first study, we adhered to the approach to measure cognitive load differentially with a questionnaire that can be answered by naïve raters without explicit knowledge of the concepts of CLT. In the second study, our aim was to develop and evaluate a questionnaire that should be able to differentiate between all three aspects of cognitive load. As the germane-load items in the previous questionnaire showed unsatisfying results for validity, we concentrated on redesigning these items first in a pilot study and afterward evaluated the overall questionnaire again in our second study, which was conducted as an online study to facilitate collecting a larger sample.

### Pilot Study for Redesigning the Germane-Load Items

To develop new, valid, and reliable items to specifically measure GCL, we conducted a pilot study where nine newly developed items for germane load were generated and tested as described in Study 1. We designed eight tasks with learning scenarios that can be expected to either result in high germane load or not. As an example, to induce GCL, we used prompts to activate learning strategies, such as asking them to write a summary of the given text or prompting them to produce a memory hook. After conducting each task, learners rated the nine new items on a 7-point-Likert-scale (from absolutely wrong to absolutely right). Twenty-seven participants took part in the pilot.

*Validity* was tested by inspecting which of the newly generated items could discriminate between low and high load tasks. A *t*-test was conducted to this end. This revealed that six out of nine items differentiated well between high and low germane-load tasks (*d* between 0.36 and 1.68 for each item). As we wanted at least two, but at most only three, items to be in the final version of the questionnaire, we decided to pick items that reflected a wide range of possible influences on GCL, that are suitable for a large variety of learning situations, and have a sufficient effect size: (1) “I made an effort, not only to understand several details, but to understand the overall context,” which reflects understanding of the overall context [*t*(26) = 4.75, *p* < 0.001, *d* = 0.94]. (2) “My point while dealing with the task was to understand everything correctly” to reflect effort of understanding everything correctly [*t*(26) = 5.31, *p* < 0.001, *d* = 0.88]. (3) “The learning task consisted of elements supporting my comprehension of the task” to reflect stimuli for deeper processing by supporting elements within the learning material [*t*(26) = 6.17, *p* < 0.001, *d* = 1.68]. The last item, which targeted supporting elements, is important for studies using worked examples, prompts for learning strategies, or similar elements that should enhance germane load. Consequently, this item may not be fitting for each learning situation but is rather important if germane load is varied on purpose. Original wording of the items in German can be found in **Table 3**.

**Table 3:** Items of the second version of the naïve rating questionnaire.

Type of load	Item - German	Item - English
ICL	Bei der Aufgabe musste man viele Dinge gleichzeitig im Kopf bearbeiten.	For this task, many things needed to be kept in mind simultaneously.
ICL	Diese Aufgabe war sehr komplex.	This task was very complex.
GCL	Ich habe mich angestrengt, mir nicht nur einzelne Dinge zu merken, sondern auch den Gesamtzusammenhang zu verstehen.	I made an effort, not only to understand several details, but to understand the overall context.
GCL	Es ging mir beim Bearbeiten der Lerneinheit darum, alles richtig zu verstehen.	My point while dealing with the task was to understand everything correct.
GCL^∗^	Die Lerneinheit enthielt Elemente, die mich unterstützten, den Lernstoff besser zu verstehen.	The learning task consisted of elements supporting my comprehension of the task.
ECL	Bei dieser Aufgabe ist es mühsam, die wichtigsten Informationen zu erkennen.	During this task, it was exhausting to find the important information.
ECL	Die Darstellung bei dieser Aufgabe ist ungünstig, um wirklich etwas zu lernen.	The design of this task was very inconvenient for learning.
ECL	Bei dieser Aufgabe ist es schwer, die zentralen Inhalte miteinander in Verbindung zu bringen.	During this task, it was difficult to recognize and link the crucial information.


*ICL, intrinsic cognitive load; ECL, extraneous cognitive load; GCL, germane cognitive load. ^∗^Item only useful if GCL is varied on purpose in a given learning material (e.g., through prompts).*

The final questionnaire for Study 2 comprised the two items for ICL and the three items for ECL already used in Study 1. Additionally, the three novel items for GCL were included. All used items (German wording and English translations) are presented in **Table 3**.

### Methods and Materials

#### Learning Tasks

For this experimental study, we created different learning tasks, which were presented online. Unlike in Study 1 (where each task was designed to vary or induce only one cognitive load type), each task varied ICL, ECL, and GCL to simulate more realistic learning situations. The variations were instantiated as follows: (a) For ICL, we varied element interactivity of a task. (b) For ECL, we presented, for example, learning environments with an integrated format of text and picture versus a separated format or just added non-relevant information. (c) For GCL, we showed learning tasks, which should either induce germane load by activating deeper learning processes versus tasks without such an activation. Experts in a pilot test have validated this classification: They had to rate for each task whether the three types of load would be high or low. Additionally, they were asked if the tasks are useful as evaluation tasks and should end up in the study or not (2 rater, Krippendorf’s α = 0.91). In the end, we decided on 17 different tasks, belonging to five different learning or problem-solving domains (vocabulary, biography, figure matching, biology, programming). For each topic, two to five tasks, varying ICL, ECL, and GCL (theoretically low versus high), were used (see Supplementary Figure S1 for details). In the vocabulary tasks, as an example, learners always had to learn three Swedish words. In one task, we used words that were similar to their German counterpart (low ICL), included unnecessary information and formatting (high ECL), but no activation was included (low GCL). In another task we used difficult words (high ICL), included memory hooks for two words and asked learners to come up with a memory hook for the third word (high GCL), but did not include unnecessary information or formatting (low ECL).

#### Participants

Between 65 and 95 participants completed each learning task and rated it. All of them were students of a German university with a major in psychology or computer science in their first or second semester. No other demographic data has been assessed.

#### Procedure

At the beginning, all participants were informed about the procedure and signed an informed consent through an online form. As participation was voluntary, participants had the chance to withdraw their data at any point in the study. All learning tasks where presented online; learners could, therefore, participate in this study without coming to a lab. After each task, learners posted their answers to the given question or their solution to the given problem and rated their perceived cognitive load with two items on ICL, three items on ECL, and three items on GCL. All items had to be answered on a 7-point Likert scale (ranging from absolutely wrong to absolutely right). The learning tasks were presented in partly random order. For some tasks, it was necessary to keep a specific order; otherwise the previous tasks would be a worked example for the preceding ones. For solving all tasks, participants needed about 45 min. Unfortunately, some participants did not conduct all tasks (65 out of 95 participants completed all tasks).

#### Data Analysis

**FIGURE 2:**
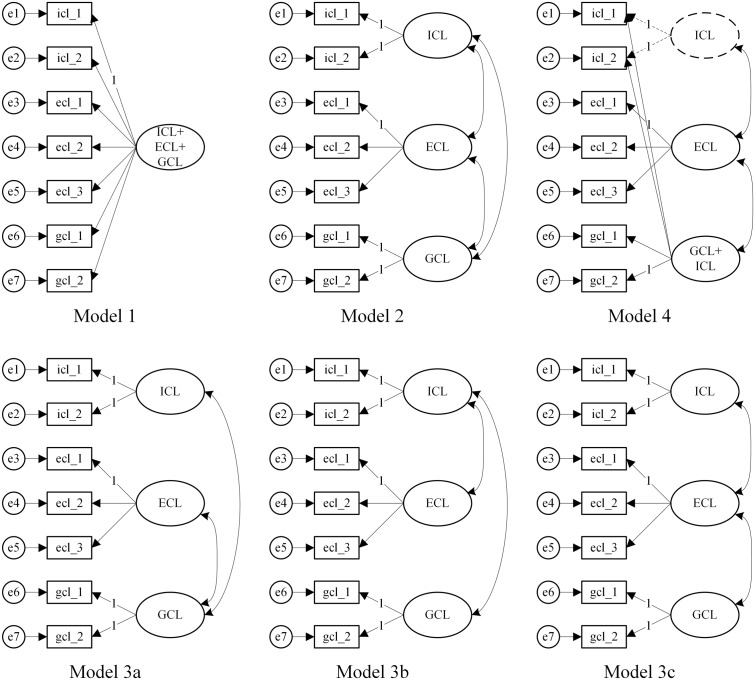
Models for running the simultaneous scenario based factor analysis.

Again, we checked *reliability* in terms of *internal consistency* per task for each type of cognitive load and reported this data in an aggregated way. The germane-load scale was analyzed with a three-item version and a two-item version, where the item for instructional support to enhance germane load within the learning task was taken out, as corresponding instructional means are not always inherent in each learning environment. If Cronbach’s alpha for the three-item GCL scale is not sufficient, all following analysis will be conducted with the two-item scale for GCL. *Validity* was analyzed like in Study 1 by testing whether the rating of the learners reflected the theoretical assumptions for each task. A confirmatory factor analysis simultaneously conducted across all tasks was used to test the *structure of the developed questionnaire.* The models, which have been tested, are shown in **Figure 2**. Model 1 assumes one unitary factor accounts for all load items. Model 2 represents the theoretical assumptions of three inter-related types of cognitive load and, therefore, suggests a three-factor model with separable but related cognitive load factors. Model 3a to 3c test if any of the latent relations between the three factors can be constrained to zero. Model 4 represents the revised perspective on cognitive load discussed by Kalyuga (2011) or Sweller et al. (2011): First, there is a productive load, comprising aspects of intrinsic and germane load, and second, there is an extraneous or unproductive load. Model 4 corresponds with this theoretical perspective: The ECL factor accounts for variance in ECL items. A broad GCL+ICL factor accounts for variance in both, GCL and ICL items. A nested ICL factor accounts for the ICL-specific variance in ICL items (i.e., technically, an ICL method specific factor). Adequate model fit is indicated by a low chi-square (χ^2^) value, a high Tucker-Lewis index (*TLI* ≥ 0.95), a high comparative fit index (*CFI* ≥ 0.95), and a low root-mean-square error of approximation (*RMSEA* ≤ 0.05). Additionally, the Akaike information criterion (*AIC*) was used as a model comparison index: The model yielding the lowest *AIC* is preferred in terms of close model fit and parsimony of the model relative to competing models.

### Results

To analyze *reliability* of the three cognitive load subscales we calculated the *internal consistency* for each task. Reliability generalization showed that the mean Cronbach’s α was 0.81 for the ICL scale, α = 0.86 for the ECL scale, and α = 0.67 for the GCL scale with three items. If the item that asked for instructional elements that support comprehension has been excluded, the remaining two-item scale for GCL resulted in an aggregated α of 0.85. If we only look at tasks that induce GCL through special elements in the learning material (e.g., prompts) the three-item version revealed an aggregated α of 0.70, as a result of more variance in the items for measuring GCL. Therefore, all following analysis were conducted using the two-item scale for GCL.

**FIGURE 3:**
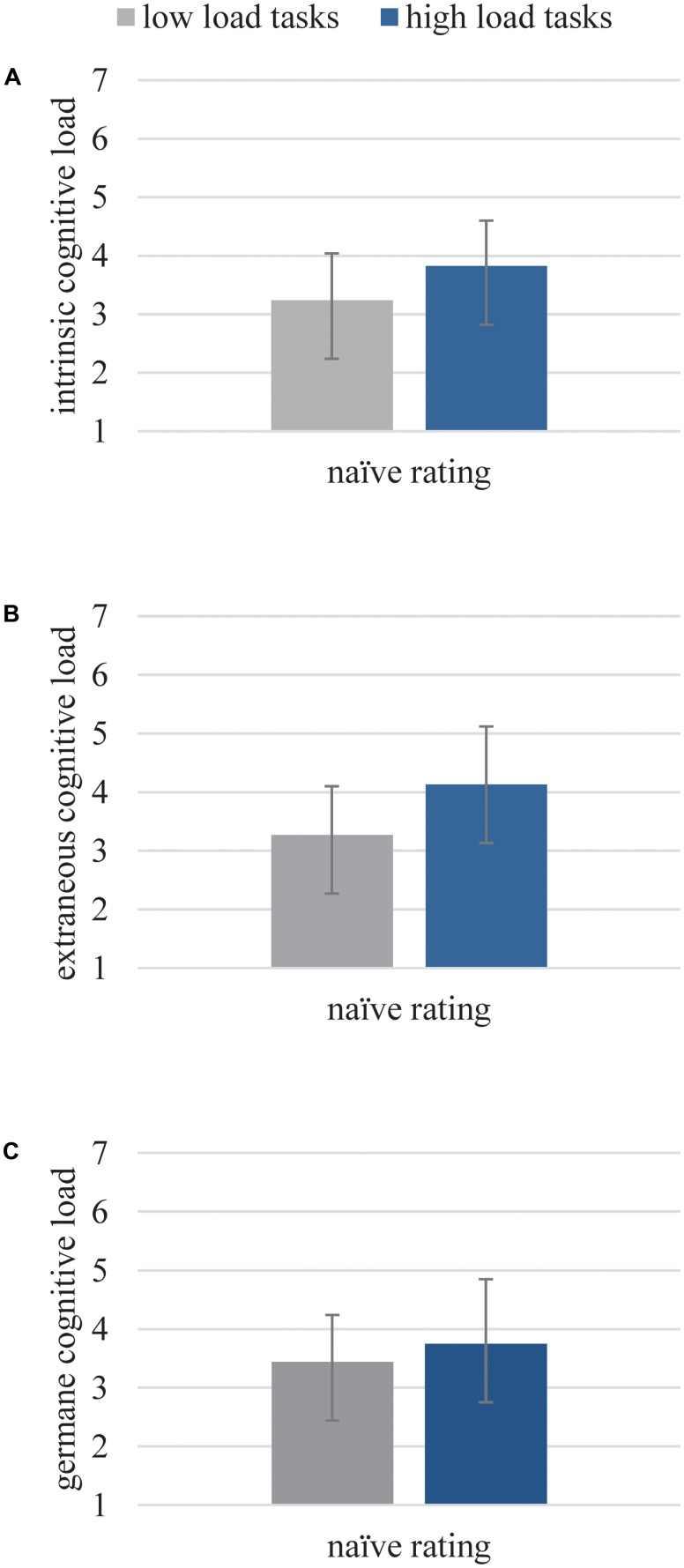
Subjective ratings of the naïve raters for ICL **(A)**, ECL **(B)**, and GCL **(C)** for corresponding theoretical low or high load tasks.

*Validity* was analyzed by comparing the ratings of the learners with the theoretically predicted outcomes, as we designed all 17 tasks to be related to high or low ICL, ECL, and GCL. Replicating results obtained in study 1, we found significantly different ratings for the low versus high load groups of tasks for each type of load [ICL: *t*(76) = 7.85, *p* < 0.001, *d* = 0.73; ECL: *t*(89) = 7.28, *p* < 0.001, *d* = 0.94; GCL: *t*(76) = 3.39, *p* < 0.01, *d* = 0.35]. Thereby, all scales were able to differentiate as expected (see **Figure 3** and **Table 4**).

**Table 4:** Means (M) and standard deviations (SD) for subjective ratings of the naïve raters for ICL, ECL, and GCL for corresponding theoretical low or high load tasks.

Type of load		Naïve raters	
		
		Low load tasks	High load tasks
ICL	*M (SD)*	3.24 (0.80)	3.82 (0.78)
ECL	*M (SD)*	3.27 (0.83)	4.13 (0.99)
GCL	*M (SD)*	3.44 (0.80)	3.75 (1.01)


*ICL, intrinsic cognitive load; ECL, extraneous cognitive load; GCL, germane cognitive load.*

As a next step, to get a closer look at the *structure of the developed questionnaire*, we conducted a confirmatory factor analysis with multiple groups. Model fits of all models can be found in **Table 5**. Overall, Model 2, representing the traditional view of three types of cognitive load, had the best fit. Conversely, Model 1 revealed the worst fit. Model 3a to 3c were inferior to Model 2. Model 4, with the recently discussed only two types of load, does reveal a decent fit. However, due to model complexity, i.e., the number of parameters to be estimated, the model comparison index AIC suggested Model 2 having the preferred fit. In conclusion, Model 2 was found to offer the best solution, thereby supporting three inter-related factors of cognitive load.

**Table 5:** Fit indices for competing structural models of cognitive load.

	χ2	*df*	*p*	*TLI*	*CFI*	*RMSEA*	*AIC*
Model 1	1709.152	238	<0.001	0.418	0.612	0.071	2,185.152
Model 2	335.106	221	<0.001	0.951	0.970	0.021	845.106
Model 3a	613.961	238	<0.001	0.851	0.901	0.036	1,089.961
Model 3b	406.207	238	<0.001	0.933	0.956	0.024	882.207
Model 3c	593.886	238	<0.001	0.859	0.906	0.035	1,069.886
Model 4	283.891	187	<0.001	0.951	0.974	0.021	861.891


### Discussion

Study 2 showed that the naïve rating with our questionnaire seems to be a promising way of assessing cognitive load. The two scales on ICL and ECL again showed satisfying reliability and validity scores, as previously shown in Study 1. After substitution of the GCL items, this scale also appeared to be sensitive as intended. Cronbach’s alpha was only moderate for the three-item version on germane load, when including one item for implemented instructional means to support learners’ activities. Internal consistency could be improved if this item were removed. However, this specific item (“The learning task consisted of elements supporting my comprehension of the task.”) is appealing because of its face validity for learning material including worked examples or prompts.

Leppink et al. (2014) discuss a similar problem, as their original four items focus on the influence of the carried-out activity. Adding one item, for invested mental effort considerably decreased internal consistency. Overall, the questionnaire had considerably good item and scale characteristics. The sensitivity of the scales to detect the theoretically implemented load variations in various testing tasks especially leads us to the conclusion that the questionnaire can be broadly used in multimedia learning research.

Concerning the question of how many load types should be captured, our confirmatory factor analysis suggested that the traditional view of three interrelated types of cognitive load factors offers the best fit for our self-report questionnaire. However, a competing model based on the current view on CLT with only two factors (and an additional item-specific method factor) followed closely, and revealed decent fit as well. Nevertheless, we prefer GCL to be considered as a separable factor because the scale was shown to reflect variation of a germane-load variation in the generated cognitive tasks. These items are especially important whenever learners are activated on purpose by instructional means. As many studies aim at analyzing such activating instructions, like using prompts or desirable difficulties, it seems of high value to measure whether learners really follow these instructions and actually engage in the learning process.

## General Discussion

The goal of our two studies was to develop a reliable, valid, and practicable instrument to measure cognitive load in a differentiated way. The current extensively used method to measure cognitive load with only one item asking for “invested mental effort” (Paas, 1992) is from a methodological view not sufficient. Furthermore, the resulting problem of not knowing which aspect of load really was enhanced and afterward inferring the possible source of load in relation to learning outcomes is in our view also not satisfying.

### Benefits of Measuring Load Differentially

With the developed questionnaire, we try to overcome the problem of inferring the source of cognitive load by using a questionnaire with several items that directly measures the constitutional parts of cognitive load, i.e., ICL, ECL, and GCL.

The differentiation can first be of interest to better understand individual learning processes. When learners with different prerequisites, such as prior knowledge, memory capacity, learning strategies, etc., deal with the same learning task, this might lead to different levels of different types of load during learning. Following, a differential approach might elect the sources of cognitive load and in addition predict learning outcome via regression analysis. Especially for comparisons of experts and novices, it can be fruitful to better understand their specific use of resources and their perception of loading sources. Furthermore, expertise reversal effects could be more easily explained and ascribed to specific types of load.

Second, the differentiated measurement can also be of great use to better understand the effects of instructional means that are not yet fully understood theoretically, like the effects of desirable difficulties. From a cognitive load point of view, one might expect that difficulties should lead to worse learning outcomes, but they actually can even enhance learning. On which type of load this fostering effect can be attributed is not yet clear but could be answered when using a differentiated load questionnaire.

We do not want to engage in the theoretical discussion of how many load types we should consider from a theoretical point of view. Instead, we argue that for *measuring* cognitive load, it can be fruitful to measure ICL, ECL, and GCL differentiated, even if the nature of germane load was questioned (Schnotz and Kürschner, 2007; de Jong, 2010; Moreno, 2010). Therefore, Ayres (2011), Kalyuga (2011), and Sweller et al. (2011) suggest rethinking the concept of GCL: It is argued that GCL, other than ICL or ECL, is not imposed by the learning material. Rather, they think that there are germane resources needed in working memory that need to be allocated to deal with the intrinsic load resulting from the learning material. There is no statement made by Sweller et al. (2011), if it is possible to allocate much more germane resources than necessary to deal with the materials’ ICL. These allocated germane resources can be used for schema acquisition and deeper understanding and additionally for elaboration and connections to prior knowledge. When thinking to an end, they are nothing other than GCL according to the traditional view (Moreno and Park, 2010) of load. However, one likes to name them, we end up measuring three types of cognitive load. In fact, this is our approach: we try to measure all processes that could require working memory resources and lead to cognitive load in some way as they are theoretically modeled in papers on CLT (e.g., Moreno and Park, 2010; Sweller, 2010a). This means that load resulting from the complexity of the learning material (especially element interactivity), load managed though prior knowledge, load as an effect of the instructional design, which could either be unproductive (e.g., split attention) or productive (e.g., prompts), and resources invested by the learner resulting in load. For all these aspects, items were created and analyzed with respect to their individual factor structure. The evaluated models in Study 2 revealed that the model with three types of load (Model 2) and the model with two types of load with the germane-load items as a part of the intrinsic-load scale (Model 4) do not differ that much, only with Model 2 resulting in minimal better model fit. Hence, from a theoretical point of view, the results provide evidence for both approaches, from a measurement point of view the three-partite model seems to be more adequate, especially when considering the reliability and validity results.

### Strengths and Weaknesses of Our Measures

Overall, the results of the presented studies imply that it is possible to measure cognitive load reliably and valid in a differentiated way. The informed rating from Study 1 especially seems to be a promising instrument to assess the different aspects of cognitive load. The downside of the informed rating strategy is that an introduction into CLT needs to be given to learners and study participants beforehand. This might result in a loss of test efficiency in a situation where the research objective is focused on testing the functionality of, e.g., a training on learning strategies. However, the method of informed rating might be an adequate instrument for analyzing cognitive aspects of instructional design of learning material and provide a promising alternative method to access the different load types occurring during working with a specific learning material. Additionally, a combination of informed and naïve rating would be a very interesting and promising way. The informed rating might be used in an early stage of learning material development. The ratings of informed experts or semi-experts, who got a standardized introduction, approve that the material is varying the intended type of load. As a next step, the naïve rating can be applied to assess the learner’s actual cognitive load during the learning process.

The naïve rating’s advantage is that it is easier to utilize and does not need as much time and cognitive investment, as no introduction to cognitive load is needed. The developed items are easy to understand and fast to answer and can be used in a variety of research projects in the field of instructional design and multimedia learning. As already mentioned, an adaption to the situation can be useful. From a methodological point of view, we must state that we did not test a huge variety of items, as is common when designing a questionnaire. This results in our top-down approach in developing our items, as mentioned above. All items reflect theoretical aspects of the different types of load. Our items were developed and carefully worded based on given definitions and descriptions of types of load through various researchers (e.g., Moreno and Park, 2010; Sweller, 2010a). Based on the results of Study 1, we state that the concepts of intrinsic and extraneous load are easy to operationalize through different items. However, Study 1 also showed us that this does not apply to the concept of GCL, mainly because of the different possibility of understanding the items. Therefore, the pilot study for Study 2 used a bottom-up approach in a first step: nine items were generated from experts. The aim was to cover various aspects of germane processes and find items that could be easily understood by learners. As a second step, we then extracted the three best-fitting items that operationalize influences on GCL by analyzing reliability and whether they differentiated well between tasks that were theoretically meant to induce either low or high levels of GCL. At this point, our recommendation for the GCL item “The learning task consisted of elements supporting my comprehension of the task” would be that it should be included based on the learning material used (whether there is instructional support for investing germane resources or not) and a test on reliability of the items on germane load to eventually exclude this item at the end of your study. We expect reliability to be good if a variation of GCL is applied on purpose in at least one group of learners in an experiment. Otherwise, reliability might be low, but it should be good if the item is excluded. Further analysis on this point is in progress.

With the newly developed differentiated questionnaire, we have to ask ourselves about the added value with respect to other existing differentiated scales, like the one from Leppink et al. (2013, 2014). Their differentiated questionnaire, in contrast to ours, is especially useful if you can clearly detect the most relevant concepts within your learning tasks, e.g., the main terms in a statistics course or a geography text. In their approach, those concepts are needed for rephrasing their items to fit the material and to analyze the load resulting from understanding these concepts. Our questionnaire is not that specific to the learning content and, therefore, only needs adoption based on the material you use, e.g., text, video, or podcast. For instance, if participants watch a learning video, it may be more appropriate to speak of “During watching this video, it was exhausting to find the important information” (instead of “During this task, it was exhausting to find the important information”). Therefore, the questionnaire is easy to apply and fits to each content, especially for short interventions like they are, e.g., analyzed in Mayers widely used multimedia learning materials (e.g., Mayer and Anderson, 1991, 1992; Mayer, 1997). Leppink et al. (2013) by themselves state that they developed the questionnaire to be used within complex knowledge domains. We developed our questionnaire to fit a wide variety of domains and studies. For longer interventions, complex learning materials, or longer learning times, we recommend using our questionnaire multiple times. This might also overcome the problem of overestimation of load as already mentioned and discussed by van Gog et al. (2011). We suggest using the questionnaire after well-selected points of time during the learning process, as it might be more meaningful when it is related to distinct parts of the learning material instead of using it after, e.g., every 10 min. We consider our questionnaire to be short enough to not interrupt learning unnecessarily.

The general problem remains that cognitive-load ratings are nevertheless self-reported measures. Learners need to be aware of their current state of cognitive resources and task demands. Regarding this, it could be interesting to add ratings of metacognitive skills to better understand learners’ abilities to self-evaluate themselves. Such skills would comprise metacognitive knowledge of one’s own memory system or of task demands and in addition the ability to monitor the learning progress during learning (Flavell, 1979). Based on their observations of the learning process learners who are skillful self-regulators might then also decide to adopt their learning behavior (Zimmerman, 1989). This naturally could influence learners perceived cognitive load. Consequently, a better understanding of learners’ self-regulatory skills could enlighten the interplay between learners’ dynamic experiences of load and their self-regulatory activities during learning as was recently discussed in a special issue on cognitive load and self-regulation (for an overview see de Bruin and Van Merriënboer, 2017).

### First Evidence of Validity and Future Directions

From a methodological point of view, we must state, that the tasks we used to impose the different types of load for validation have been of short duration and were embedded in an artificial learning context. Therefore, we started to evaluate the questionnaire with a study program where we explicitly varied ICL, ECL, and GCL in real learning settings with greater samples. This approach additionally has the advantage that learning outcomes can be derived. Overall, with these studies we aim to prove the validity of our instrument. Again, for the design of these studies, we used a highly systematic approach with varying domains in which only one type of load is addressed to be varied on purpose. ICL should be varied especially in terms of element interactivity, whereas ECL variations should be covering a wide variety of multimedia principles. For GCL, a variation of instructional help through, e.g., prompts, should be considered. Also, variations covering motivation and personal involvement seem to be of value to better understand the interplay of motivational or affective states and cognitive load. First results are promising in terms of a good sensitivity of our instrument regarding the intended load variations—in type and level—as the two following studies demonstrate.

A study of Seufert et al. (2016) already used our questionnaire in an experiment on increasing disfluency levels. Their main result was that slightly to medium levels of disfluency can foster learning compared to a fluent text, but that learning outcome gets worse when disfluency gets too intense. With respect to cognitive load, they found no effects on intrinsic load as expected. However, extraneous load was on a medium level for all disfluency groups, despite the group with the highest level of disfluency, which has been rated as highly loading extraneously. Germane load, which was called engagement in their study, on the other hand, increases steadily with increasing disfluency and remains high even on the highest disfluency level. Nevertheless, this engagement on the highest disfluency level does not pay off, as extraneous load is also too high. This is especially interesting as germane load—against theoretical assumptions—in this case, is related to low learning outcomes. Only when taking all aspects of load into account, can one understand the whole picture. Leppink et al. (2014) decided to drop their scale on GCL because they did not find an acceptable correlation between learning outcome and invested germane load. In our view, this positive relation is not *per se* to be expected as the interplay between different load types; the overall amount of load and especially the individual skills affect whether enhanced germane processes really are successful.

Another study using the presented naïve questionnaire was conducted by Rogers et al. (2014). They investigated the different systems for learning piano. The developed system, P.I.A.N.O., with its novel roll notation directly onto the piano keys, should avoid split attention and motivate learners through fast learning success. It resulted in the most accurate performance while playing a piece of music compared to a group using a standard sheet notation and a group using the software Synthesia to learn the piece of music. The projected roll notation also reduced perceived intrinsic load significantly and the therefore avoided split attention also reduced extraneous load. The novel roll notation also resulted in significant higher germane load, than in the group with the standard sheet notation. In a user experience test, they also found P.I.A.N.O. to be ranked highest among their tested systems. Based on GCL ratings and the measured user experience, Rogers et al. (2014) assume a motivational factor to be relevant. Motivation is also stated as important to learning and cognitive load by Zander (2010). Zander shows that motivational prerequisites of learners influence cognitive load: If a learner is motivated, allocation of resources in working memory to deal with ICL, ECL, and GCL is appropriate for task difficulty. If a learner is not motivated, fewer resources are allocated to deal with cognitive load. If resources are limited already through insufficient motivation, ICL and ECL might block all available resources, so nothing more is left for GCL. Unfortunately, Zander tried, but wasn’t successful, in measuring cognitive load in a differentiated way. A replication of her study with our naïve rating would be a great possibility to get more insights into the relationship between cognitive load and the motivational prerequisites of learners.

What’s next? For further analyses and improvement, the naïve rating questionnaire will be implemented and tested in different learning situations and with a variation of multimedia effects and domains as mentioned above. This should provide further evidence that our questionnaire is sensitive to different variations of different load types and, therefore, valid. With such a reliable, valid, and easy-to-use measure, which is used in many different studies with many different learning tasks and learner types, we should be able to learn more about the nature of cognitive load during learning and hence to theoretically improve CLT in the long run.

## Ethics Statement

This study was exempt from an ethic committee approval due to the recommendations of the German Research Association: All subjects were in no risk out of physical or emotional pressure, we fully informed all subjects about the goals and process of this study and none of the subjects were patients, minors or persons with disabilities. In all studies participation was voluntary and all subjects signed a written informed consent and were aware that they had the chance to withdraw their data at any point of the study.

## Author Contributions

MK and TS contributed to the conception and design of the studies. MK developed the used material and questionnaires and led data collection for all studies. TS revised the questionnaires. MK and FS analyzed and interpreted the data. MK drafted the work, which was revised critically by FS and TS. All authors provided approval of the final submitted version of the manuscript and agree to be accountable for all aspects of the work in ensuring that questions related to the accuracy or integrity of any part of the work are appropriately investigated and resolved.

## Conflict of Interest Statement

The authors declare that the research was conducted in the absence of any commercial or financial relationships that could be construed as a potential conflict of interest.

## Footnotes

1 As the logic of classical significance testing is inverted when testing for the absence of differences, we adopted a more conservative criterion. Thereby, we sought to reduce a possible beta error, i.e., assuming no differences despite there are some in reality.
