# Supplementary material for: Development and Validation of Two Instruments Measuring Intrinsic, Extraneous, and Germane Cognitive Load
Source: Front Psychol. 2017 Nov 16;8:1997. doi: 10.3389/fpsyg.2017.01997 (PMC5696680; doi:10.3389/fpsyg.2017.01997)
Supplement: Supplementary file 1 [file Table_1.PDF]

Supplementary Material

Development and Validation of two Instruments measuring Intrinsic, Extraneous, and Germane Cognitive Load

Melina Klepsch*, Florian Schmitz, Tina Seufert

*** Correspondence:** Melina Klepsch: melina.klepsch@uni-ulm.de

1 Evaluation Tasks of Study 1

Supplementary Table S1 contains all evaluations tasks of Study 1 and information on the cognitive load which has been varied. The tasks are in German, therefore, a description of the task is included.

Supplementary Table S1. Evaluation tasks and their intended cognitive load variation.

type of load	evaluation tasks: low load tasks		evaluation tasks: high load tasks	description
ICL	Merke dir folgende Vokabeln<br>• Tante – Aunt<br>• Onkel – uncle<br>• Oma - grandma		Merke dir folgende schwedische Wörter<br>Baum – träd<br>Auto – bil<br>Ochse - gnor	remember vocabulary
	Berechne das Ergebnis<br>$\frac{5}{9}+\frac{4}{9}=$		Berechne das Ergebnis<br>$\frac{\frac{3}{4}*\left(\frac{5}{9}+\frac{4}{9}\right)*\frac{6}{8}}{\frac{6}{8}*\left(\frac{1}{4}+\frac{2}{4}\right)}=$	solving fractions
	Von welchem Tag wird gesprochen?<br>Gestern war Dienstag,<br>welcher Tag ist übermorgen		Von welchem Tag wird gesprochen?<br>Angenommen der 5. Tag<br>nach dem gestrigen Tag war Mittwoch,<br>welcher Tag ist dann morgen?	guess the correct day
	Wie alt ist Peter?<br>Frau Schmid ist heute 60 Jahre alt.<br>Ihr Sohn Peter ist halb so alt wie sie.		Wie alt ist Frau Köpernick?<br>Frau Köpernick ist heute doppelt<br>so alt wie ihr Sohn Tom.<br>Vor 10 Jahren war sie dreimal so alt wie dieser.	calculate someones age
ECL	Befindet sich in der Lungenschlagader sauerstoffreiches oder sauerstoffarmes Blut?<br>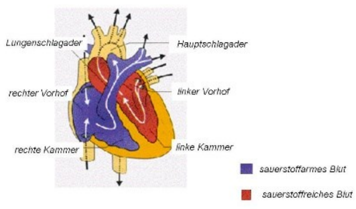	(1)	Befindet sich in der rechten Kammer sauerstoffreiches oder sauerstoffarmes Blut?<br>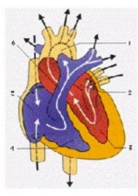<br>Fluß des Blutes durch das menschliche Herz, blau bedeutet sauerstoffarmes, rot bedeutet sauerstoffreiches Blut<br>1= Hauptschlagader (Aorta)<br>2= linker Vorhof<br>3= linke Kammer<br>4= rechte Kammer<br>5= rechter Vorhof<br>6= Lungenschlagader	funcionality of the human heart
	Warum sind einmal 2 und einmal 3 P zu sehen?<br>Die Bildung von ATP geschieht in 3 Schritten:<br>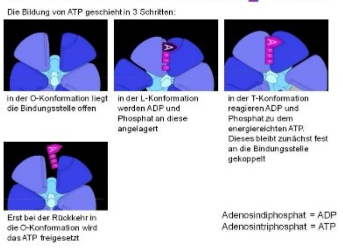Adenosindiphosphat = ADP<br>Adenosintriphosphat = ATP	(2)	Warum ist in einem Bild eine Lücke zwischen den P ?<br>Adenosindiphosphat = ADP<br>Adenosintriphosphat = ATP<br>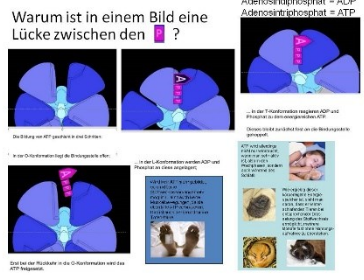	understand ATP synthase
	Welches Ventil ist offen, welches geschlossen?<br>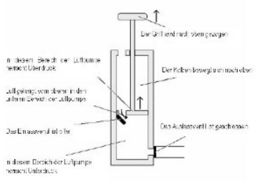	(3)	Wo herrscht Überdruck? Wo Unterdruck?<br>Funktionsweise einer Luftpumpe<br>Eine Luftpumpe besteht aus einem Kolben, der innerhalb eines Gehäuses von oben nach unten bewegt werden kann. Außerdem sind zwei Ventile notwendig, damit die Luftpumpe ihre Funktion erfüllen kann: ein Einlass- und ein Auslassventil.<br>Zieht man den Griff des Kolbens nach oben, bewegt dieser sich nach oben, wodurch im oberen Teil der Pumpe Überdruck, im unteren Unterdruck entsteht. Da das Einlassventil in dieser Phase geöffnet ist, strömt aufgrund dieses Druckunterschiedes Luft vom oberen in den unteren Teil der Pumpe. Damit die Luft dort nicht sofort entweichen kann, ist das Auslassventil geschlossen.<br>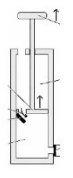	function of a bicycle tire inflator
	Was ist $0{,}1^2$?<br>Gebe zwei gleichwertige Antworten!<br>http://www.youtube.com/watch?v=PG7yQ8eq5Pk&feature=related<br>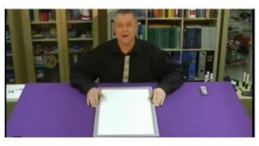<br>Erst den Film anschauen, dann antworten...	(4a) (4b)	Wann ist a^0 nicht 1?<br>http://www.youtube.com/watch?v=LHuZxgBoQrg<br>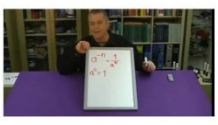<br>Hallo, jetzt kommt eine Definition auf die sich jeder Lehrende hoffentlich freut, denn die zieht immer Widerspruch nach sich, diese Definition, und ist daher immer sehr interessant.<br>Also, wir haben das Potenzieren mit negativen Exponenten, und zwar geht es um a^{-n}<br>n soll hier eine natürliche zahl sein und dann ist –n eine negative Zahl. Das ist ein a. Und a^{-n} ist gleich 1 geteilt durch a^n<br>Und wenn wir schon mal dabei sind kann man auch gleich noch festlegen was a0 ist. A^0 ist gleich 1. Egal was man für a einsetzt, außer 0, aber ansonsten ist a^0 immer 1	understand power in mathematics
	Wie viele Teile mit der Nummer 101201 solltest du haben?<br>Teil / Teilenummer / Anzahl<br>112996 – 1x<br>113434 – 8x<br>101351 – 8x<br>101532 – 8x<br>101201 – 16x<br>106996 – 4x	(5)	Wie viele Teile mit der Nummer 101201 solltest du haben?<br>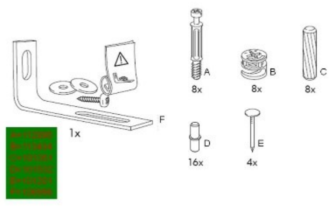	count parts to assemble furniture
GCL	• Stellen Sie sich vor Sie hören einen Vortrag ohne Folien, ohne Visualisierung und ohne Denkanregungen. Der Dozent liest lediglich einen Text ab, der zwar klar und eindeutig verständlich ist, aber eben sehr lang.		• Ein Dozent nennt zu Beginn der Vorlesung klare Lehrziele: er beschreibt, was Sie am Ende wissen sollen und erläutert Ihnen warum das wichtig ist.	
	• Sie sind es gewohnt am Ende einer Vorlesung so genannte to-go-messages aufzuschreiben, damit Sie nicht gleich wieder alles Wichtige vergessen. Der Dozent lässt Ihnen aber weder zwischendurch noch am Ende Zeit dazu und Sie müssen leider auch gleich weiter zur nächsten Vorlesung.		• Der Dozent unterbricht seine Vorlesung hin und wieder mit sogenannten Chatgroups<br>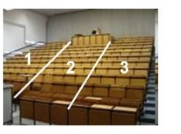	imagine classroom situations
	• Sie hören eine Vorlesung in Englisch und können selbst nicht besonders gut englisch sprechen oder verstehen.		• Eine Dozentin bringt für ihre schwierigen Themen immer wieder Beispiele. Immer nach etwa 20 Minuten lässt sie Sie selbst jeweils ein Beispiel finden oder eine konkrete Anwendungsaufgabe durcharbeiten.	

(1) Schmidt T. (1995). *Einführung in die Physiologie des Menschen*. Springer, Berlin.
(2) Seufert, T., Schütze, M. & Brünken, R. (2009). Memory characteristics and modality in multimedia learning: An aptitude-treatment-interaction study. *Learning and Instruction*, 19, 28-42.
(3) Mayer, R. & Anderson, R. (1991). Animations need narrations: An experimental test of a dual-coding hypothesis. *Journal of Educational Psychology*, 83, 484-490.
(4a) hannaholb (2009, April 17) *Potenzen üben negative Dezimalzahlen 1* [Video file]. Retrieved from https://www.youtube.com/watch?v=PG7yQ8eq5Pk
(4b) hannaholb (2009, April 17) *Potenzen – Definition von negative Exponenten* [Video file]. Retrieved from http://www.youtube.com/watch?v=LHuZxgBoQrg
(5) IKEA (2010). *Billy*
