# Supplementary material for: Development and Validation of Two Instruments Measuring Intrinsic, Extraneous, and Germane Cognitive Load
Source: Front Psychol. 2017 Nov 16;8:1997. doi: 10.3389/fpsyg.2017.01997 (PMC5696680; doi:10.3389/fpsyg.2017.01997)
Supplement: Supplementary file 2 [file Image_1.PDF]

Supplementary Material

Development and Validation of two Instruments measuring Intrinsic, Extraneous, and Germane Cognitive Load

Melina Klepsch*, Florian Schmitz, Tina Seufert

*** Correspondence:** Melina Klepsch: melina.klepsch@uni-ulm.de

2 Variation in tasks of Study 2

Supplementary Figure S1 contains the clustering of domain, tasks and cognitive load type variation of Study 2, to provide evidence, that all possibly permutations in load type variation have been included.

	ICL	ECL	GCL
**Vocabulary**	Task 01 (high)<br>Task 09 (low)<br>Task 14 (low)<br>Task 16 (low)	Task 01 (low)<br>Task 09 (high)<br>Task 14 (low)<br>Task 16 (low)	Task 01 (low)<br>Task 09 (low)<br>Task 14 (high)<br>Task 16 (high)
**Biography**	Task 02 (high)<br>Task 04 (low)<br>Task 06 (high)<br>Task 10 (low)<br>Task 12 (high)	Task 02 (high)<br>Task 04 (high)<br>Task 06 (low)<br>Task 10 (low)<br>Task 12 (low)	Task 02 (low)<br>Task 04 (high)<br>Task 06 (high)<br>Task 10 (low)<br>Task 12 (high)
**Figure matching**	Task 03 (high)<br>Task 07 (low)	Task 03 (low)<br>Task 07 (high)	Task 03 (low)<br>Task 07 (high)
**Biology (cells)**	Task 05 (high)<br>Task 11 (low)	Task 05 (high)<br>Task 11 (low)	Task 05 (low)<br>Task 11 (high)
**Java-programming**	Task 08 (low)<br>Task 13 (low)<br>Task 15 (low)<br>Task 17 (high)	Task 08 (high)<br>Task 13 (low)<br>Task 15 (low)<br>Task 17 (high)	Task 08 (low)<br>Task 13 (low)<br>Task 15 (high)<br>Task 17 (low)

Supplementary Figure S1. Clustering of domain, tasks and cognitive load type variation.
